# Case Report: Primary vascular sarcoma presenting as massive pulmonary embolism salvaged by veno-arterial extracorporeal membrane oxygenation and interventional therapy

**DOI:** 10.3389/fcvm.2026.1765928

**Published:** 2026-03-19

**Authors:** Jinmei Huang, Peiyi Lin, Liting Wang, Pingping Xiao, Wei Fan, Lihong Tian, Yi Zhang, Shufan Wu, Zhigao Dong

**Affiliations:** 1 Department of Rheumatology and Immunology, The Second Affiliated Hospital of Xiamen Medical College, Xiamen, China; 2 Department of Interventional Vascular Surgery, The Second Affiliated Hospital of Xiamen Medical College, Xiamen, China; 3 Department of Pathology, The Second Affiliated Hospital of Xiamen Medical College, Xiamen, China

**Keywords:** acute massive pulmonary embolism, case report, ECMO, interventional thrombectomy, primary vascular-derived sarcoma, thrombolytic

## Abstract

**Introduction:**

Acute massive pulmonary embolism (massive PE) caused by primary vascular-derived sarcoma is extremely rare in clinical practice. Massive PE results in severe shock or cardiac arrest, with high mortality and adverse clinical progression. We report the case of a young woman with a primary vascular-derived sarcoma-related massive PE, diagnosed through aspiration biopsy performed during angiography. The patient was successfully treated using veno-arterial extracorporeal membrane oxygenation (VA-ECMO) combined with interventional thrombectomy, thrombolysis, and anticoagulation.

**Case description:**

A 34-year-old woman experienced 2 days of right thigh pain and acute dyspnea, culminating in transient syncope (3 h). On admission, she was hemodynamically unstable, with markedly elevated D-dimer levels. Computed tomography angiography confirmed a massive PE and extensive deep vein thrombosis involving the right iliac and femoral veins. Within the first hour after admission, she experienced cardiorespiratory arrest, requiring immediate initiation of VA-ECMO. Combined therapy (interventional thrombectomy, thrombolysis, and anticoagulation) stabilized cardiopulmonary function, allowing successful weaning from VA-ECMO. Venography revealed a filling defect from the right common iliac vein into the inferior vena cava. Pathological examination of an aspirated thrombus specimen revealed the presence of atypical cells. Immunohistochemistry supported the diagnosis of primary vascular-derived sarcoma (angiosarcoma). Following clinical stabilization, she was discharged on therapeutic rivaroxaban and scheduled for systemic chemotherapy for angiosarcoma.

**Conclusions:**

This case illustrates that VA-ECMO, combined with interventional thrombectomy, thrombolysis, and anticoagulation, is an effective salvage therapy for angiosarcoma-associated massive PE, particularly in patients with hemodynamic instability. It highlights the need to suspect occult malignancy, including vascular sarcomas, in young patients with unprovoked massive PE. Moreover, aspiration biopsy during angiography, when clinically indicated, can serve as a critical diagnostic tool.

## 1 Introduction

Acute massive pulmonary embolism (massive PE) is a syndrome characterized by obstruction of the pulmonary arterial system by large endogenous or exogenous emboli. Its primary clinical and pathophysiological manifestations include right heart dysfunction, hemodynamic instability, and hypoxemia (1). Massive PE represents the most severe form of pulmonary embolism, with mortalities ranging from 25.0% to 52.4% (2, 3). Although thrombolysis is currently the mainstay treatment for massive PE, its efficacy is time-dependent and often uncertain, resulting in treatment failures in some patients and inevitably heightens the risk of bleeding complications, including life-threatening hemorrhage. As an alternative measure, extracorporeal membrane oxygenation (ECMO) provides robust respiratory and circulatory support, rapidly stabilizes vital signs, and offers unique advantages in managing massive PE.

Massive PE caused by primary vascular-derived sarcoma is exceedingly rarely encountered in clinical practice. In this report, we describe the case of a young female patient with a primary vascular-derived sarcoma-related massive PE, who was successfully treated using veno-arterial extracorporeal membrane oxygenation (VA-ECMO) combined with interventional thrombectomy, thrombolysis, and anticoagulation.

## 2 Case description

A 34-year-old woman had experienced right thigh pain for 2 days, with dyspnea and a transient (3 h) loss of consciousness. Her vital parameters indicated hemodynamic instability: hypotension (84/50 mmHg), hypoxemia (SpO_2_ 89%), core hypothermia (35.0 °C), sustained tachyarrhythmia (135 bpm), and profound tachypnoea (46 breaths/min). She appeared drowsy and restless, with clammy skin and dyspnea. Her right thigh was thicker than the left, and the dorsalis pedis artery was palpable. The patient had no history of medical comorbidities, hereditary disorders, or chronic substance use (alcohol/nicotine).

The laboratory profile on admission was as follows: hematologic parameters included D-dimer levels of 18,408 ng/mL (high increase; NR < 500); the cardiac profile revealed a cardiac troponin I level of 1.745 ng/mL (NR < 0.016) and a B-type natriuretic peptide level of 111.1 pg/mL (NR < 100); and acid–base testing revealed a pH of 7.05 (NR 7.35–7.45), a base excess of −21.6 mmol/L (NR −3–3), and a lactate level of 12.21 mmol/L (NR 0.5–2.0).

**Figure 1:**
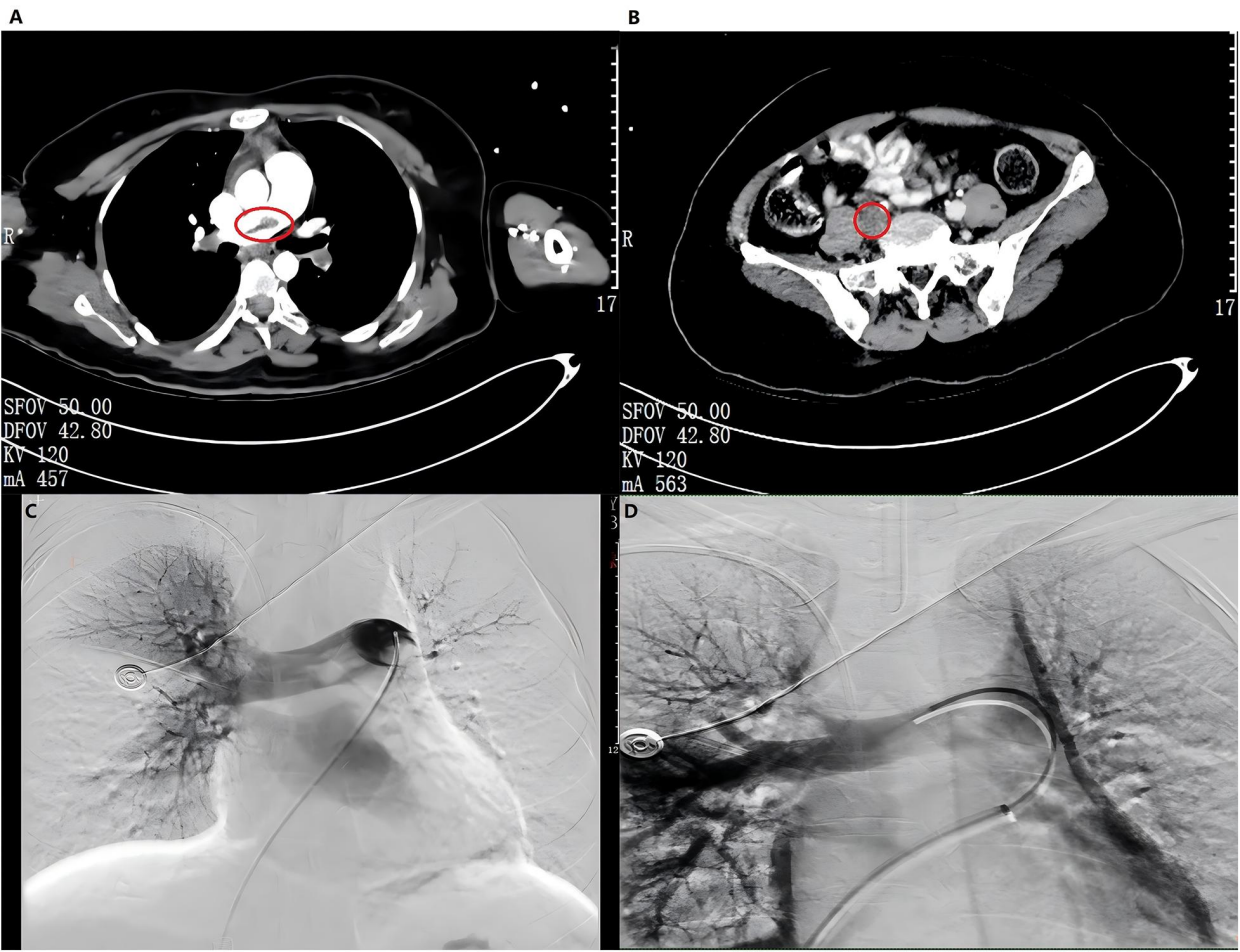
Abdominal/chest computed tomography scans and angiography of the patient. **(A)** Multiple filling defects in the main pulmonary artery, bilateral pulmonary trunk, and partial pulmonary branches of both lower lobes of the lungs: extensive pulmonary embolism was considered. **(B)** Multiple filling defects in the right common iliac vein, right internal and external iliac vein, and femoral vein: thrombosis was considered. **(C)** The angiographic manifestations before interventional thrombectomy. **(D)** The angiographic manifestations after interventional thrombectomy.

Abdominal contrast-enhanced computed tomography revealed multiple filling defects in the main pulmonary artery, bilateral pulmonary trunk, and partial pulmonary branches of both lower lobes of the lungs, suggesting extensive pulmonary embolism (Figure 1A). Multiple filling defects were also observed in the right common iliac vein, right internal and external iliac veins, and the femoral vein, suggestive of thrombosis (Figure 1B). Echocardiography revealed right ventricular dilation (right/left ventricle diameter ratio of 1.3), a reduced left ventricular size, a tricuspid annular plane systolic excursion of 9 mm, moderate tricuspid regurgitation, and an estimated pulmonary artery systolic pressure of 40 mmHg. The patient was administered oxygen, fluid rehydration, and a combination of norepinephrine with dopamine to increase the blood pressure and correct acidosis, in addition to 50 mg alteplase for thrombolysis.

Within the first hour after admission, the patient experienced respiratory and cardiac arrest, for which cardiopulmonary resuscitation, emergency medications, and mechanical ventilation were initiated. However, shortly after achieving spontaneous circulation after 6 min of cardiopulmonary resuscitation, the patient developed gradual recurrent bradycardia. Given the continued instability of her breathing and circulation, VA-ECMO support was immediately commenced. Following VA-ECMO, pulmonary angiography and thrombolysis were performed in the intervention room, and several subacute thrombi were aspirated with approximately 100 mL of blood. The branch arteries at all levels of both lungs were established to have undergone significant enlargement, accompanied by a marked increase in the blood flow velocity (Figures 1C, D). Follow-up echocardiography subsequent to ECMO therapy revealed a reduction in the right ventricular size (right/left ventricle diameter ratio 0.9), normalization of the left ventricular dimensions, a tricuspid annular plane systolic excursion of 18 mm, mild tricuspid regurgitation, and an estimated pulmonary artery systolic pressure of 23 mmHg.

On day 3 after admission, the patient's breathing and circulation had stabilized, ECMO was withdrawn, and an inferior vena cava filter was inserted. On day 6 after admission, the tracheal tube was removed, and 3 days later, the patient underwent lower limb venous catheterization and iliac vein balloon angioplasty; following surgery, she was treated with heparin anticoagulation and urokinase thrombolytic therapy. The thrombolytic process was successful.

**Figure 2:**
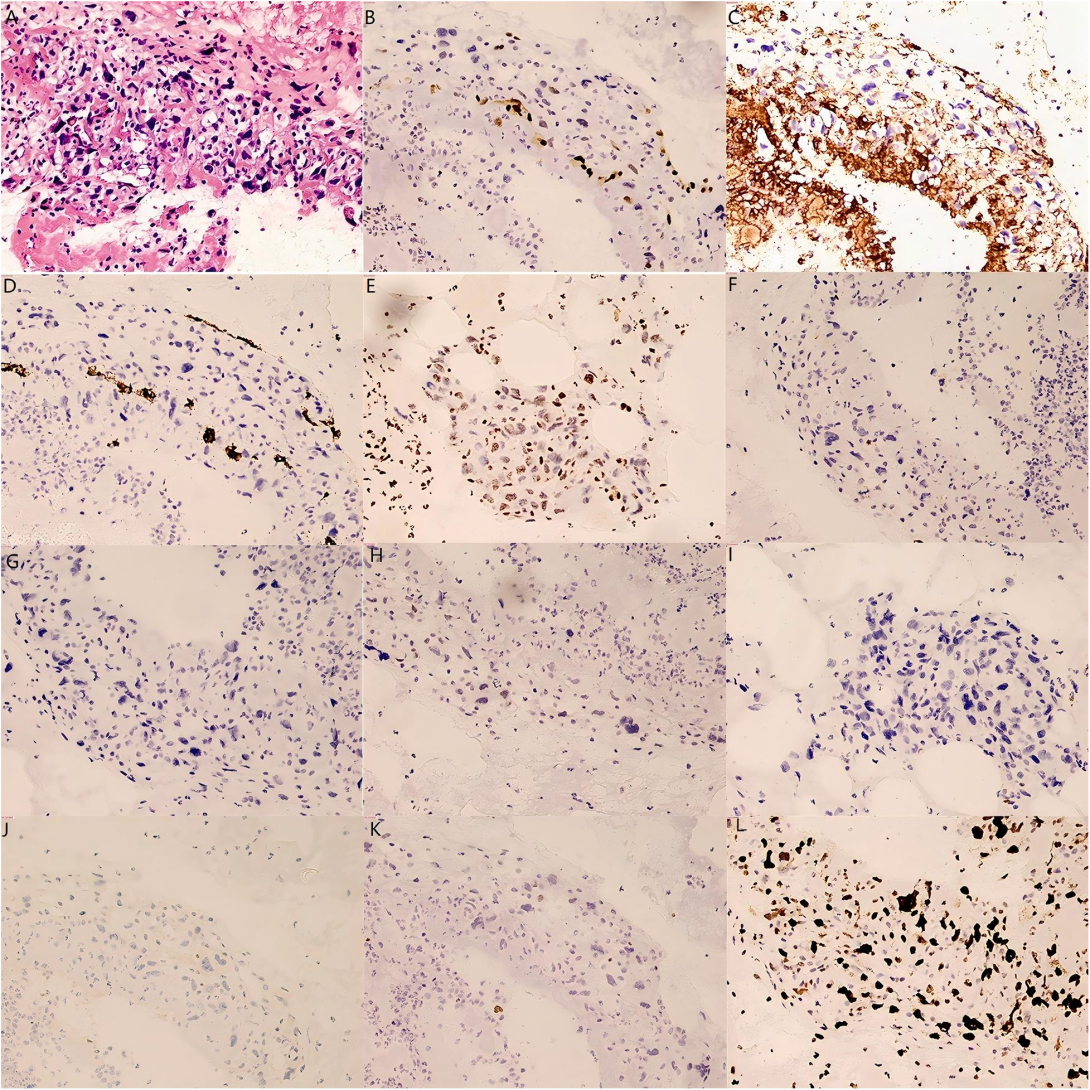
The pathological and immunohistochemical results of the patient's thrombus tissue. **(A)** HE X200, a small amount of atypical cells were observed in the thrombus tissue of the patient; **(B)** ERG X100(Focal +); **(C)** CD31 X200(+); **(D)** CD34 X200(rare +); **(E)** Fli-1 X200(Focal +); **(F)** CK X200(-); **(G)** PAX-8 X200(-); **(H)** SALL-4 X200(-); **(I)** CD30 X200(-); **(J)** ER X100(-); **(K)** GATA-3 X200(-); **(L)** Ki-67 X200(40%+).

On day 12, repeat venography of the lower limbs revealed that the proximal filling defect of the right common iliac vein was regular in shape and protruded approximately 4 cm into the inferior vena cava. A small tissue sample was aspirated for pathological examination, a popliteal vein puncture sheath was inserted, and heparin was administered as part of the anticoagulation therapy. Examination of the tissue sample revealed a small number of atypical cells (Figure 2A) and, combined with immunohistochemical findings (ERG [focal +], CD31 [+], CD34 [rare +], FLI-1 [focal +], CK [–], PAX-8 [–], SALL [–], CD30 [–], ER [–], GATA-3 [–], and Ki-67 [40% +]), supported a provisional diagnosis of primary vascular-derived sarcoma (Figure 2).

On day 18, the patient's symptoms had improved, and her condition had stabilized. She was discharged on rivaroxaban for anticoagulation and referred to a specialized center for systemic chemotherapy. She received six cycles of a combination chemotherapy regimen of epirubicin and ifosfamide regimen Q3W (epirubicin 60 MG/M2 d1, ifosfamide 1.8 G/M2 d1-3). In addition, treatment included the administration of granulocyte colony-stimulating factor and mesna. Six months following discharge, she remained in good general condition and continued oral anticoagulation and scheduled chemotherapy treatment. The timeline of major clinical and nursing events is presented in Table 1.

**Table 1:** Timeline of Major clinical and nursing events.

Time since Admission	Clinical Event/Status	Nursing & Medical Interventions
Day 0 (Admission)	Presentation with hemodynamic instability (hypotension, hypoxemia, hypothermia, tachyarrhythmia), dyspnea, altered mental status, and right thigh pain	Initial Stabilization: Administered oxygen, fluid resuscitation. Vasoactive Support: Initiated combination norepinephrine and dopamine infusion. Thrombolysis: Administered 50 mg alteplase. Monitoring: Continuous vital sign and neurological status monitoring.
Hour 1 (Day 0)	Respiratory and cardiac arrest	Emergency Response: Immediate initiation of cardiopulmonary resuscitation (CPR), emergency medications, and endotracheal intubation with mechanical ventilation. ROSC achieved after 6 min of CPR.
Post-ROSC (Day 0)	Recurrent gradual bradycardia with unstable breathing and circulation. Abnormal echocardiographic parameters	Advanced Support: Immediate initiation of Veno-Arterial Extracorporeal Membrane Oxygenation (VA-ECMO) for cardiopulmonary support.
Day 0 (Post-ECMO)	On ECMO support. Imaging confirmed extensive PE and DVT	Interventional Procedure: Transfer to interventional room for pulmonary angiography, thrombolysis, and thrombus aspiration. Post-procedure: Continued intensive monitoring on VA-ECMO.
Day 3	Breathing and circulation stabilized. Significant improvement in echocardiogram parameters.	Weaning from Support: Successful withdrawal of VA-ECMO. Prophylaxis: Insertion of an inferior vena cava (IVC) filter to prevent further embolism.
Day 6	Respiratory function improved	Liberation from Ventilator: Tracheal extubation performed.
Day 9	Ongoing management of deep vein thrombosis (DVT)	Surgical Intervention: Underwent lower limb venous catheterization and iliac vein balloon angioplasty. Pharmacological Therapy: Post-operative initiation of therapeutic heparin anticoagulation and urokinase thrombolytic therapy.
Day 12	Re-examination of lower limb venous system. Identification of an unusual intravascular mass	Diagnostic Procedure: Venography and aspiration of a small tissue sample from the iliac vein filling defect for pathological examination. Therapy: Continued anticoagulation with heparin.
Day 18	Symptoms improved, condition stabilized. Patient felt well.	Discharge Planning: Patient discharged from hospital. Education & Coordination: Prescribed rivaroxaban for anticoagulation. Referred to a specialized oncology center for further diagnosis and management of suspected primary vascular-derived sarcoma.
6 Months Post-Discharge	Follow-up visit	Outcome: Patient in acceptable general condition. Ongoing Care: Continuing scheduled chemotherapy and oral anticoagulant therapy.

DVT, deep vein thrombosis; ECMO, extracorporeal membrane oxygenation; IVC, inferior vena cava; PE, pulmonary embolism; ROSC, return of spontaneous circulation.

## 3 Discussion

Massive PE secondary to primary vascular-derived sarcoma is exceptionally rare and presents with nonspecific symptoms. Such tumour-associated embolic events are generally more severe and urgent than conventional thromboembolic episodes, and the underlying malignancy often remains undiagnosed until advanced clinical deterioration. The risk of pulmonary embolism increases with several well-established factors, including the use of estrogen-containing oral contraceptives, pregnancy, advanced age, obesity, smoking, trauma, systemic infection, prolonged immobility or paraplegia, autoimmune disorders (e.g., antiphospholipid syndrome), and inherited thrombophilias (e.g., Factor V Leiden mutation, protein C/S deficiency, antithrombin deficiency) (4, 5). This clinical presentation of an unprovoked, catastrophic embolism in a young individual initially raised suspicion of a potential non-thrombotic etiology, such as a tumor embolism, rather than a simple, bland thrombus.

Before pathological confirmation, the differential diagnosis for this young woman with acute massive PE and extensive deep vein thrombosis (DVT) included several non-neoplastic possibilities. The primary consideration was a “thrombus-in-transit” originating from the right lower extremity. However, the absence of typical risk factors and the disproportionate thrombotic burden prompted evaluation of alternative etiologies. Second, “bland thrombus with reactive endothelial atypia” represented a crucial, albeit rare, differential diagnosis. Ischemic or mechanical stress can cause entrapped cells to mimic malignancy cytologically. This highlights why diagnosis relies on immunohistochemistry (e.g., ERG, CD31) to define cell lineage and exclude this benign mimic.

In the context of tumor-related embolism, primary pulmonary artery sarcoma (PPAS) should be considered in the differential diagnosis. PPAS is a rare malignancy that frequently mimics pulmonary thromboembolism clinically and radiologically. Key diagnostic challenges in differentiating PPAS from PTE include: overlapping symptoms (dyspnea, chest pain), elevated D-dimer (which is also common in PPAS), and imaging similarities on CT angiography (6–8). However, certain features may raise suspicion for PPAS, such as a filling defect expanding the pulmonary artery, heterogeneous enhancement on contrast-enhanced CT, uptake on positron emission tomography-CT, and the presence of concomitant thrombus (which often coexists with tumor) (8–10). As highlighted in a recent review (9), distinguishing PPAS from PTE requires a high index of suspicion, especially in patients with “unprovoked” embolism, and often hinges on histopathological confirmation. In our case, the tumor originated from the iliac vein rather than the pulmonary artery, but the diagnostic challenges and importance of tissue sampling remain analogous.

In this patient, multiple clinical and imaging features suggested a possible neoplastic origin. First, the magnitude and rapid progression of the thrombotic events, including extensive iliofemoral DVT leading to near-fatal PE, appeared disproportionate to the absence of traditional prothrombotic risk factors. Second, follow-up venography on day 12 revealed a well-defined, protruding filling defect (approximately 4 cm) in the proximal right common iliac vein extending into the inferior vena cava. This lesion exhibited a regular, solid morphology atypical of a simple, organizing thrombus, which often appears irregular or serpiginous. Table 2 summarizes the distinguishing features between conventional PE and tumor embolism, underscoring the importance of considering malignancy in atypical presentations (7, 8, 11–13).

**Table 2:** Distinguishing features: conventional pulmonary embolism vs. tumor embolism.

Feature	Conventional Pulmonary Embolism	Tumor Embolism (e.g., Vascular Sarcoma)
Clinical Context	Provoked (risk factors present) or unprovoked	Often unprovoked in young patients; rapid, catastrophic presentation
Clinical Onset	Acute, may be recurrent	Can be subacute or rapidly progressive; symptoms may precede embolism
Thrombus Burden	Variable, may correlate with risk factors	Disproportionately massive and rapidly progressive
Imaging (CT/Venography)	Filling defect often irregular, may be multiple. Chronic thrombus may cause vessel retraction	Filling defect may be expansive, smooth-contoured, distending the vessel. It may show contrast enhancement
D-dimer	Typically, very high in acute setting	Can be very high, not discriminatory
Response to Anticoagulation/Thrombolysis	Clinical and radiological improvement expected	Often poor or transient; rapid recurrence of the “thrombus”
Definitive Diagnosis	Clinical/imaging correlation; thrombus pathology reveals bland fibrin and platelets	Histopathology of embolic material (atypical/malignant cells)

Aspiration biopsy performed during interventional angiography was pivotal in establishing the definitive diagnosis. Tissue acquisition was pursued due to a high pre-test probability of malignancy and the unique opportunity provided by the therapeutic thrombectomy. Although not a standard component of routine thrombectomy for presumed bland PE, targeted biopsy of suspicious, refractory, or morphologically atypical clot material can provide an opportunistic and innovative approach to identifying obscure embolic sources. In this case, after hemodynamic stabilization with VA-ECMO and initial thrombectomy, we aspirated a small tissue sample from the distinctive iliac vein lesion under fluoroscopic guidance. This approach carries potential risks, including vessel injury, perforation, or dislodgement of additional tumor material; however, in a controlled setting by an experienced interventional team, the additional risk over the therapeutic procedure is expected to be minimal. The successful acquisition of diagnostically viable tissue highlights the value of integrating diagnostic sampling into the therapeutic workflow for complex, unexplained PE.

The diagnostic criteria (14) for primary angiosarcoma include: (1) a histological morphology consistent with the characteristics of malignant vascular endothelial cell tumours; (2) immunohistochemistry confirming vascular endothelium differentiation, with at least two positive markers (CD31 and ERG being the most specific, and CD34/FLI1 as supportive markers); and (3) exclusion of other morphologically similar malignancies. In the current case, negative staining for epithelial, germ cell, breast, urothelial, and melanocytic markers ruled out other tumour types, while positivity for endothelial markers (ERG, CD31, CD34, and FLI-1) supported the diagnosis of primary vascular-derived sarcoma.

Massive PE often causes profound shock or cardiac arrest, with high early mortality (approximately 10% of patients die within the first hour of symptom onset). In such critical cases, VA-ECMO can serve as a stand-alone therapy or as a bridge to more advanced therapies (15). In our patient, VA-ECMO was initiated as a rescue therapy following recurrent hemodynamic collapse after initial thrombolysis and cardiopulmonary resuscitation. According to the 2019 European Society of Cardiology Guidelines for the diagnosis and management of acute PE and the Pulmonary Embolism Response Team consensus, VA-ECMO is indicated as a salvage therapy in patients with refractory cardiac arrest or shock despite conventional measures and may serve as a bridge-to-reperfusion or bridge-to-decision tool (16, 17). Thrombolysis aims to pharmacologically recanalize the pulmonary arteries and improve right ventricular function; however, its effect is delayed and not uniformly effective, which can lead to circulatory collapse. Immediate VA-ECMO initiation offers three key benefits: it bypasses the obstructed pulmonary circulation, reduces venous return—thereby reducing right ventricular wall tension and preload—and creates a therapeutic window for subsequent catheter thrombectomy, thrombolysis, and anticoagulation (18–20). Given that thrombolysis can dislodge deep vein thrombi and cause recurrent embolism, placement of an inferior vena cava filter prior to thrombolysis is recommended (21). Overall, ECMO can serve as an important tool for saving patients with acute cardiopulmonary failure, with reported cardiac-support survival rates of approximately 41% (22, 23).

Regarding oncologic management, pathological confirmation of pulmonary tumor emboli established a diagnosis of metastatic (Stage IV) angiosarcoma. The therapeutic goal thus shifted to systemic disease control. The multidisciplinary team deferred radical primary tumor resection for three reasons: 1) the inability of surgery to manage distant metastases; 2) the high operative risk associated with iliac vein/inferior vena cava location in the context of acute cardiopulmonary failure; and 3) the established role of systemic chemotherapy as the primary treatment for Stage IV disease. A first-line epirubicin-ifosfamide regimen was initiated for palliative cytoreduction, disease control, and embolism risk reduction. Although no other primary tumor site was identified on subsequent imaging, the prognosis of primary vascular sarcoma presenting with embolic disease remains guarded, with reported median survival often less than 12–18 months despite aggressive treatment (8, 24). At 6-month follow-up, the patient was clinically stable on combined rivaroxaban and chemotherapy (six cycles completed), underscoring the value of a coordinated multimodal approach.

Long-term management of sarcoma-related pulmonary embolism requires a careful balance between anticoagulation efficacy and the risks associated with bleeding. In the current case, anticoagulation was transitioned from low-molecular-weight heparin to rivaroxaban, consistent with guideline recommendations for cancer-related pulmonary embolisms (25, 26).

In conclusion, the present case demonstrates the effectiveness of combining VA-ECMO with interventional thrombectomy, thrombolysis, and anticoagulation as salvage therapy for sarcoma-related massive PE, particularly in hemodynamically unstable patients. Successful implementation requires multidisciplinary coordination, including critical care, cardiology, oncology, and interventional radiology teams. This case highlights the need to suspect occult malignancy, including vascular sarcomas, in young patients with unprovoked massive PE. In addition, aspiration biopsy during angiography, when clinically indicated, can serve as a critical diagnostic tool. Further research is needed to validate the efficacy and safety of this combined approach in sarcoma-specific populations.

## Data Availability

The original contributions presented in the study are included in the article/Supplementary Material, further inquiries can be directed to the corresponding author.
